# Serological Assessment of Enzootic Status and Associated Risk Factors for Cattle Tick Fever in Herds from Paraíba State, Northeast Brazil

**DOI:** 10.3390/pathogens15090932

**Published:** 2026-09-04

**Authors:** Felipe Boniedj Ventura Alvares, Jordania Oliveira Silva, Basilio Felizardo Lima Neto, Geraldo Moreira Silva Filho, Thais Ferreira Feitosa, Vinícius Longo Ribeiro Vilela

**Affiliations:** 1 Postgraduate Program in Science and Animal Health, Federal University of Campina Grande—UFCG, Patos 58055-018, PB, Brazil; felprathalos@gmail.com (F.B.V.A.); jordaniaoliveira6091@gmail.com (J.O.S.); felizardobasilio95@gmail.com (B.F.L.N.); geraldosfilho4@gmail.com (G.M.S.F.); 2 Veterinary Parasitology Laboratory, Department of Veterinary Medicine, Federal Institute of Paraíba—IFPB, Sousa 58805-345, PB, Brazil; thais.feitosa@ifpb.edu.br

**Keywords:** anaplasmosis, babesiosis, risk factor, seroprevalence

## Abstract

Cattle tick fever (CTF) is the most important tick-borne disease complex affecting cattle production in tropical and subtropical regions. This study evaluated the enzootic status of CTF in cattle herds from two contrasting climatic regions of Paraíba State, Northeast Brazil, and identified epidemiological factors associated with herd immune status. A cross-sectional serological survey was conducted in 32 cattle herds from two contrasting climatic regions of Paraíba State. All herds were tested for *Babesia bovis* and *Babesia bigemina*, whereas 28 herds (14 per region) were evaluated for *A. marginale*. Herds were classified according to the proportion of seropositive animals for each pathogen as non-immune (≤12.5%), unstable (>12.5% to <75%), or stable (≥75%), and epidemiological factors associated with enzootic status were subsequently investigated using logistic regression models. Antibodies against at least one pathogen were detected in 55.4% (97/175) of the evaluated cattle, with higher overall seroprevalence in the Tropical Humid region (56/85; 65.9%) than in the Semi-arid region (41/90; 45.6%). Antibodies anti-*B. bovis* showed the lowest prevalence (24/175; 13.7%), whereas anti-*A. marginale* (60/155; 38.7%) and anti-*B. bigemina* (58/175; 33.1%) were more frequently detected. No herd was classified as enzootically stable for anti-*B. bovis*, whereas 6/32 (18.8%) and 3/28 (10.7%) herds achieved enzootic stability for anti-*B. bigemina* and anti-*A. marginale*, respectively. Herds composed predominantly of cattle older than five years were 97% less likely to develop protective seropositivity against *B. bovis*. Dairy production and animal purchase increased the odds of protective seropositivity against *B. bigemina* by approximately 74-fold and 10-fold, respectively, whereas needle reuse reduced the likelihood of protective seropositivity against *A. marginale* by 97%. These findings demonstrate that enzootic instability predominates in cattle herds from Paraíba State, indicating that natural pathogen circulation alone has been insufficient to establish consistent herd immunity against the cattle tick fever pathogens.

## 1. Introduction

Cattle production represents one of the most important agricultural activities in Brazil, playing an important role as a food source and in national agribusiness performance [1]. In the Northeast region, cattle farming is predominantly conducted under extensive or semi-extensive systems, frequently exposed to environmental stressors such as high temperatures, irregular rainfall, and seasonal feed scarcity [2,3]. These conditions directly influence animal health and productivity, increasing vulnerability to infectious and parasitic diseases [4,5].

Among the diseases that most strongly affect cattle health in tropical and subtropical regions, cattle tick fever (CTF) stands out as a disease complex that is caused by three intraerythrocytic pathogens: *Babesia bovis*, *Babesia bigemina*, and *Anaplasma marginale* [6,7,8]. While *Babesia* spp. are protozoa transmitted biologically by *Rhipicephalus microplus*, *A. marginale* is a rickettsial bacterium with a broader epidemiological profile, as it can be transmitted not only by ticks but also mechanically by hematophagous flies and iatrogenically during routine veterinary procedures [9].

The economic impact of CTF is substantial, and the losses are associated not only with acute clinical disease and mortality but also with subclinical infections that lead to reduced weight gain, lower milk yield, reproductive inefficiency, and increased veterinary costs [10]. In Brazil, parasitic diseases associated with ticks are estimated to cause annual losses exceeding billions of dollars, largely due to productivity decline and expenses related to chemical control [11,12]. Additionally, the intensive use of acaricides has contributed to the emergence and spread of *R. microplus* populations resistant to multiple chemical classes, further complicating disease control and altering the epidemiological balance of CTF pathogens [8,13].

In a previous study carried out by Alvares et al. [7], high PCR positivity rates were observed across different climatic regions of Northeast Brazil, with particularly elevated detection in the tropical humid region and substantial circulation also in semi-arid areas. These findings confirm widespread pathogen circulation in the region, but they do not allow an interpretation of the enzootic status of CTF in the studied regions, as molecular detection only reflects active infection, not the immunological history of the herds.

The distinction between enzootic stability and instability is important for CTF epidemiology. In enzootically stable areas, early and continuous exposure of calves to the pathogens results in widespread immunity, reducing the occurrence of severe clinical disease. On the other hand, enzootic instability is characterized by irregular exposure, which leads to partial immunity and a higher risk of outbreaks affecting susceptible animals [14]. In regions such as the semi-arid areas of the State of Paraíba, where climatic conditions may limit vector activity during part of the year, high PCR positivity raises important questions regarding the real epidemiological equilibrium of the disease [7].

Although PCR detection is highly sensitive for identifying infected animals, it does not indicate whether exposure has occurred early and consistently enough to establish protective herd immunity within the population. Consequently, PCR findings alone cannot determine whether cattle herds are enzootically stable or remain susceptible to clinical outbreaks. Serological evaluation is therefore essential because it reflects previous pathogen exposure and allows the classification of herds according to their enzootic status.

Therefore, this study aimed to characterize the enzootic status of cattle tick fever in cattle herds from the semi-arid and tropical humid regions of Paraíba State through serological evaluation. By classifying herds according to their immune status and identifying epidemiological factors associated with enzootic stability, this study provides epidemiological information that contributes to a more comprehensive understanding of CTF dynamics in Northeast Brazil.

## 2. Materials and Methods

### 2.1. Study Area

The study was conducted in Paraíba State, the Northeast region of Brazil, encompassing two distinct climatic zones, the Semi-arid and the Tropical Humid regions. These areas present marked environmental differences that may influence the ecology of *R. microplus* and the epidemiology of CTF.

The Semi-arid region, located in the western portion of Paraíba, is characterized by a hot semi-arid climate (BSh, Köppen classification), with a well-defined rainy season concentrated between February and May, followed by a prolonged dry season extending from June to January [15]. Annual rainfall ranges from 400 to 800 mm, with average annual temperatures of 28 °C, low thermal amplitude, and mean relative humidity around 60%, showing marked seasonal variation [16].

The Tropical Humid region of Paraíba, located mainly along the eastern coastal zone, presents substantially higher annual rainfall, ranging from approximately 1200 mm to over 1800 mm, with persistently high relative humidity throughout the year. Average annual temperatures are 26 °C, with minimal seasonal variation [17].

### 2.2. Study Design and Sampling Strategy

A cross-sectional epidemiological survey was conducted to evaluate the enzootic status of CTF in cattle herds from the Semi-arid and Tropical Humid regions of the State of Paraíba. Field sampling was conducted from February to July 2023. A two-stage sampling strategy was adopted for multistage sampling in veterinary epidemiological surveys [18].

Farms were considered the primary sampling units, whereas individual cattle represented the secondary sampling units. Herds containing at least 5 cattle were eligible for inclusion in the study. The selected farms predominantly represented small-scale family production: 93.8% (30/32) were family farms, and 87.5% (28/32) had 40 cattle or fewer.

Within each selected herd, five animals were randomly sampled from herds containing up to 20 cattle, whereas six animals were randomly sampled from herds with more than 20 cattle. This sampling strategy was designed to maximize the probability of correctly classifying the infection status of each herd while increasing the geographical representativeness of the survey through the inclusion of a larger number of herds. This approach is consistent with multistage epidemiological sampling, in which animals sampled within the same herd are expected to share similar management practices, environmental conditions, and pathogen exposure and therefore do not constitute statistically independent observations [18].

**Figure 1:**
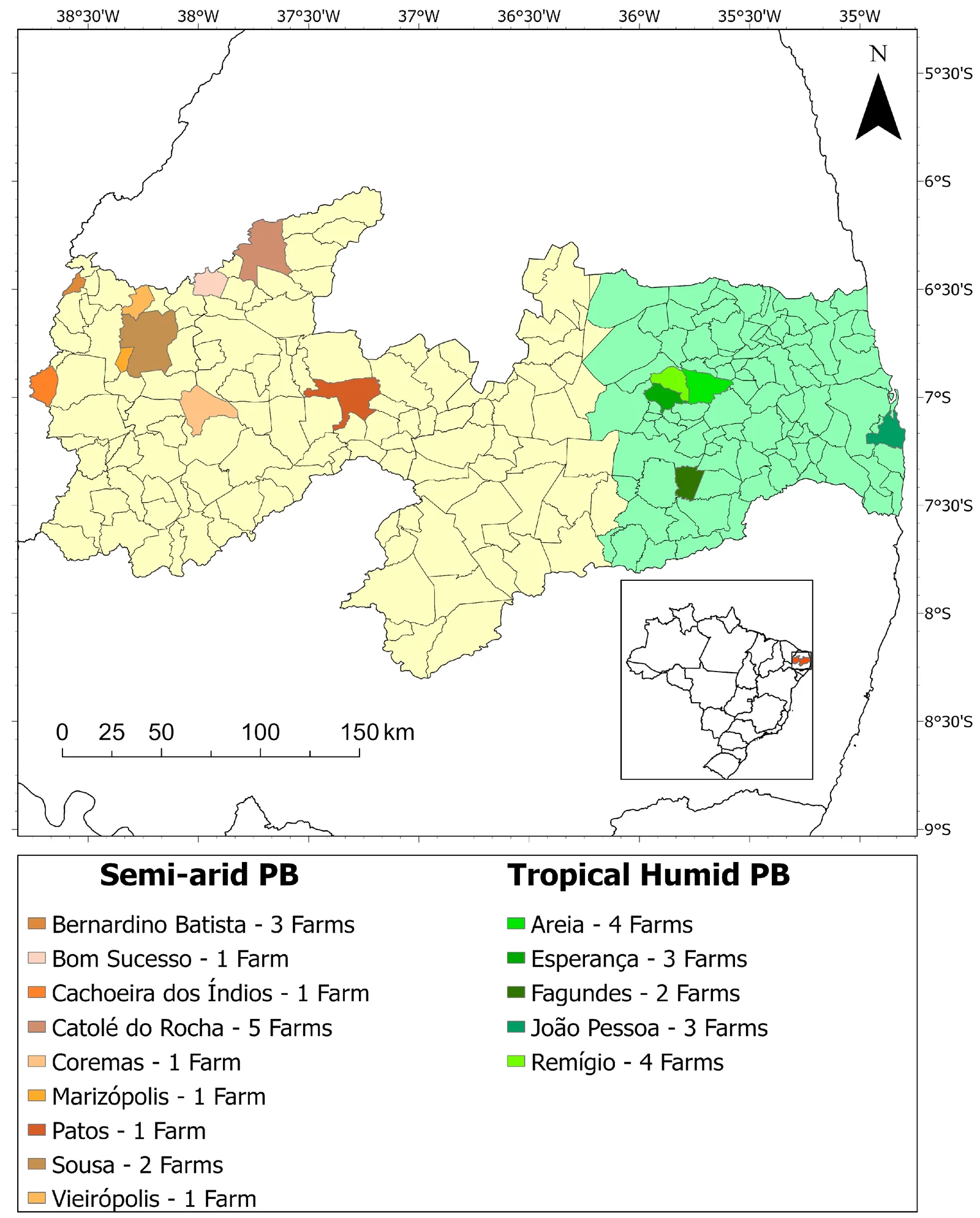
Geographic distribution of sampled cattle farms in Semi-arid and Tropical Humid regions of the State of Paraíba, Northeast Brazil.

A total of 175 serum samples from cattle belonging to 32 farms, 16 distributed across the Semi-arid (*n* = 90 animals) and 16 in the Tropical Humid (*n* = 85 animals) regions of Paraíba, were included in the serological analyses (Figure 1).

### 2.3. Blood Sample Processing

Blood samples were collected by jugular or caudal venipuncture using sterile vacuum tubes without anticoagulant. Samples were maintained at 2–8 °C during transportation and processed within 24 h after collection.

Serum samples were obtained by centrifugation at 1500× *g* for 15 min. Approximately 1.5 mL of serum from each animal was transferred into sterile 2.0 mL microtubes, prepared in duplicate, properly identified, and stored at −20 °C until serological analysis.

### 2.4. Serological Analysis (ELISA)

The presence of IgG antibodies against *B. bovis* and *B. bigemina* was determined using commercial indirect enzyme-linked immunosorbent assay (ELISA) kits based on crude antigen preparations (Bovine Babesia bovis ELISA Kit, AFG Scientific, Northbrook, IL, USA; Cattle Babesia bigemina ELISA Kit, AFG Scientific, Northbrook, IL, USA). Antibodies against A. marginale were detected using an iELISA kit containing partially soluble antigen (Imunoteste^®^—Anaplasma marginale ELISA, bovine, Laboratórios, SP, Brazil). All serum samples were tested in duplicate, with the corresponding positive and negative controls included in each assay.

Because the *A. marginale* assay required a greater number of control wells and blank wells per plate, fewer samples could be accommodated in each run. Given the limited number of available kits, only 28 herds could be evaluated for *A. marginale*. Four herds were therefore randomly selected for exclusion, with two herds from each region, and all sampled animals within the 28 selected herds were tested. Thus, 155 animals from 28 herds were evaluated for *A. marginale*, whereas all 175 animals from 32 herds were tested for *Babesia* spp.

Sample classification was performed according to the cut-off criteria specified by the manufacturers. For the *B. bovis* and *B. bigemina* ELISA kits, the cut-off was calculated as the mean optical density (OD) of the negative control plus 0.15. Samples with OD values equal to or above the calculated cut-off were considered positive. For the *A. marginale* ELISA, the cut-off index was calculated as 2.5 times the OD of the negative control, with samples presenting OD values equal to or above this index considered positive.

The ELISAs were qualitative. The manufacturer of the *A. marginale* ELISA reports a diagnostic sensitivity >83% and specificity >81%; corresponding diagnostic sensitivity and specificity estimates were not reported in the available manufacturer documentation for the *B. bovis* and *B. bigemina* ELISA kits. Reactions were read using a microplate ELISA reader (LMR-96i, Loccus, Cotia, SP, Brazil), and sample classification was performed automatically by the reader according to the cutoff criteria established by the respective manufacturers. No manual interpretation of absorbance values was performed.

### 2.5. Epidemiological Questionnaire

Information on herd management and epidemiological practices was obtained through a structured questionnaire administered to each cattle producer. The questionnaire addressed herd characteristics, including cattle population, breed, production system (intensive, extensive, or semi-intensive), production purpose (dairy or beef), and grazing area. Information regarding animal movement and contact patterns was also collected, including animal acquisition, contact with other cattle herds, shared pasture use, and bull rotation between farms for reproductive purposes.

The questionnaire further addressed health-related and management practices potentially associated with CTF transmission. Producers were asked about the main health problems observed on the farm and previous CTF cases, as well as the reuse of needles following applications. The presence of hematophagous flies, including horse flies (Tabanidae), stable flies (*Stomoxys calcitrans*), and horn flies (*Haematobia irritans*), was also recorded.

### 2.6. Statistical Analysis

Statistical analyses were performed using R software (R Core Team, Vienna, Austria). Initially, descriptive statistics were calculated to determine the frequency of seropositive animals for *A. marginale*, *B. bovis*, and *B. bigemina* in each climatic region. The enzootic status of each farm was subsequently classified as Non-immune (≤12.5%), Unstable (>12.5–<75%), or Stable (≥75%), following the serological classification adopted by Lagranha et al. [19], based on the concept of enzootic stability proposed by Mahoney and Ross [20].

The epidemiological unit adopted for the regression analyses was the farm. Epidemiological variables obtained from the questionnaire were considered as potential explanatory variables in the risk-factor analyses. Associations between epidemiological variables and farm enzootic status were initially evaluated by univariate analyses. Variables presenting *p* ≤ 0.20 were considered eligible for inclusion in the multivariable models, following the recommendations of Hosmer and Lemeshow [21].

Whenever no farms were classified as Stable, Unstable or Non-immune for a pathogen of the CTF complex, the outcome consisted of only two categories. Therefore, binary logistic regression models with binomial error distribution and logit link function were fitted to evaluate factors associated with the enzootic status of this pathogen.

Whenever the enzootic status comprised three ordered categories (Non-immune, Unstable, and Stable), proportional odds ordinal logistic regression models were fitted to evaluate the association between epidemiological variables and enzootic status while preserving the natural ordering of the response variable.

Odds ratios (OR), 95% confidence intervals (95% CI), and corresponding *p*-values were estimated for all explanatory variables retained in the final models. Statistical significance was considered at *p* ≤ 0.05. No formal adjustment for multiple comparisons was applied, as the risk-factor analyses were exploratory and followed a predefined univariate screening step (*p* ≤ 0.20) before construction of the multivariable models.

## 3. Results

Among the 175 sampled cattle, 30.3% (53/175) were aged 0–2 years, 45.7% (80/175) were aged 2–5 years, and 24.0% (42/175) were older than 5 years. Females accounted for 81.7% (143/175) of the sampled animals, while males accounted for 18.3% (32/175). Regarding breed, 6.9% (12/175) of the cattle were Holstein, 78.3% (137/175) were Holstein crossbred, and 14.9% (26/175) were Zebu.

Antibodies against at least one CTF pathogen were detected in 97 out of 175 cattle evaluated (55.4%). Overall seroprevalence was higher among animals from the Tropical Humid region (65.9%; 56/85) than among those from the Semi-arid region (45.6%; 41/90) (Table 1).

Seroprevalence against *B. bigemina* showed the greatest variation between climatic regions, with 22.2% in the Semi-arid region and 44.7% in the Tropical Humid region. In contrast, antibodies against *A. marginale* were detected at similar percentages in both regions (38.7% and 38.6%, respectively), whereas *B. bovis* exhibited the lowest seroprevalence, 13.3% and 14.1%, respectively (Table 1).

**Figure 2:**
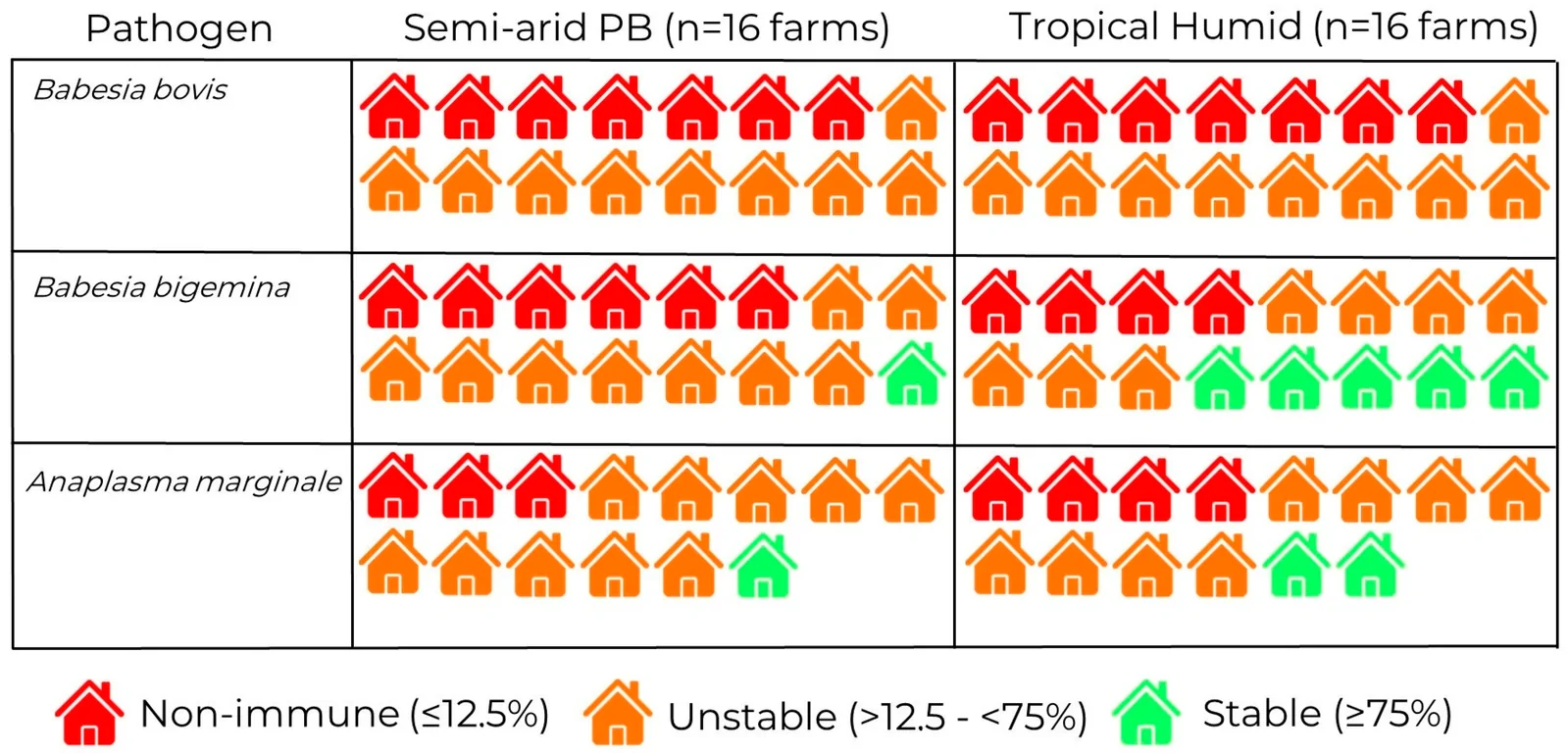
Distribution of farms according to enzootic status for each CTF pathogen in the Semi-arid and Tropical Humid regions of Paraíba. Each icon represents one evaluated farm.

The enzootic status differed markedly among the evaluated pathogens (Figure 2). No farm achieved seroprevalence above 75% for *B. bovis* and, consequently, no one was classified as enzootically Stable for this pathogen. Conversely, 14 (43.8%) farms were classified as Non-immune, indicating that nearly half of the evaluated herds presented seroprevalence below the 12.5% threshold adopted for enzootic classification.

*Babesia bigemina* presented the highest number of enzootically Stable farms, particularly in the Tropical Humid region, where five out of 16 evaluated farms were classified as Stable. In contrast, only one Stable farm was identified in the Semi-arid region, while *A. marginale* showed three Stable farms distributed between both climatic regions. Despite these differences, the Unstable category predominated for all three pathogens, accounting for 50 (16/32) to 56.2% of the farms (18/32).

During the univariate screening, age and shared pasture fulfilled the selection criterion (*p* ≤ 0.20) and were included in the multivariable model. However, after adjustment for potential confounding factors, only age remained significantly associated with the enzootic status of *B. bovis* (Table 2).

Farms composed predominantly of animals older than five years showed markedly lower odds of achieving stable enzootic status than those with cattle aged 0–2 years (OR = 0.03; 95% CI: 0.00–0.44; *p* = 0.025).

Although shared pasture met the inclusion criterion during the univariate analysis (*p* = 0.138), its association with the enzootic status was no longer observed after multivariable adjustment (*p* = 0.207). Likewise, the comparison between cattle aged 2–5 and 0–2 years remained non-significant in both analyses.

During the univariate screening, region, age, breed, production type, animal purchase, and bull rotation between farms fulfilled the inclusion criterion (*p* ≤ 0.20). After adjustment for potential confounding factors, only breed, production type, and animal purchase remained independently associated with enzootic stability (Table 3).

Dairy farms showed markedly greater odds of achieving enzootic stability than beef farms (OR = 74.51; 95% CI: 3.80–6760; *p* = 0.017). Likewise, farms composed predominantly of Holstein cattle exhibited greater odds of enzootic stability than those composed predominantly of Zebu or other breeds.

Notably, breed became statistically significant only after multivariable adjustment. Although none of the breed categories reached statistical significance during the univariate analysis, Holstein cattle showed significantly greater odds of enzootic stability after adjustment for potential confounding factors.

During the univariate screening, age, animal purchase, bull rotation between farms, and reuse of needles met the selection criterion (*p* ≤ 0.20) and were included in the multivariable model. Among the evaluated variables, reuse of needles was the only factor independently associated with the enzootic status of *A. marginale* after multivariable analysis (Table 4). Farms reporting the reuse of needles showed markedly lower odds of achieving stable enzootic status (OR = 0.03; 95% CI: 0.00–0.38; *p* = 0.019).

Although bull rotation between farms showed a strong association in the univariable analysis (OR = 11.19; *p* = 0.039), this association was no longer significant after multivariable adjustment (*p* = 0.125). Likewise, animal purchase and age lost statistical significance in the final model.

## 4. Discussion

Serological evaluation revealed that 55.4% of the evaluated cattle had antibodies against at least one cattle tick fever (CTF) pathogen. However, despite this relatively high seroprevalence, marked differences were observed in cattle immunity among the three pathogens, indicating that pathogen exposure alone does not necessarily result in enzootic stability. When interpreted alongside the PCR detection rates of 82.7% for at least one CTF pathogen previously reported for the same cattle population [7], these findings demonstrate that widespread pathogen circulation and herd immunity represent distinct epidemiological components.

Enzootic stability is not determined by the simple presence of pathogens within a herd but by the continuous and early exposure of susceptible calves, allowing immunity to develop before clinical disease occurs [20,22]. Consequently, herds with intense pathogen circulation may still remain enzootically unstable when exposure is insufficient, irregular, or delayed. The contrasting stability profiles observed among the three pathogens reinforce this concept.

Climatic conditions are among the main determinants of enzootic stability because they directly influence the population dynamics of *R. microplus*, the principal biological vector of CTF pathogens in Brazil. Temperature, relative humidity, and rainfall affect tick survival and the number of generations produced annually, resulting in marked regional differences in host exposure [16,23]. In tropical humid environments, these conditions favor the maintenance of tick populations throughout most of the year, whereas prolonged droughts in semi-arid regions [15] reduce larval survival and limit parasite transmission [24].

### 4.1. Enzootic Status of B. bovis

No herd achieved enzootic stability for *B. bovis*, despite previous molecular evidence demonstrating pathogen circulation in both climatic regions [7]. This finding represents the most concerning epidemiological scenario observed in the present study, indicating that exposure was insufficient to establish protective herd immunity at the population level. Because *B. bovis* is recognized as the most pathogenic agent of the CTF complex [24,25], low herd immunity substantially increases the risk of severe clinical outbreaks following the introduction of infected ticks into susceptible herds.

The complete absence of enzootically stable herds is biologically plausible considering the transmission dynamics of *B. bovis*. Compared with *B. bigemina*, *B. bovis* generally produces lower parasitemia, reducing the likelihood of parasite acquisition by feeding ticks and consequently limiting transmission efficiency within cattle populations [25]. This reduced transmission pressure probably contributes to irregular exposure of susceptible calves, making the establishment of herd immunity more difficult, particularly in regions where *R. microplus* infestation is seasonal or occurs at low intensity [22,26].

Age was the only variable independently associated with the enzootic status of *B. bovis* in the multivariable model. Herds composed of cattle older than five years exhibited 97% lower odds of presenting enzootically Stable profiles (OR = 0.03), reinforcing the importance of early exposure for the establishment of protective herd immunity. This association should not be interpreted as evidence that older cattle are intrinsically less capable of developing immunity against *B. bovis*. Instead, it probably reflects insufficient or delayed exposure during the first years of life, when calves are naturally more resistant to clinical disease and are expected to acquire protective immunity under enzootically stable conditions [20,25].

Consequently, the predominance of older animals in herds with less favorable enzootic profiles suggests that natural immunization failed to occur at the appropriate age, increasing the likelihood of enzootic instability. Strategic tick control could produce prolonged periods of enzootic instability and increase the risk of babesiosis outbreaks, illustrating how reductions in tick-mediated transmission may interrupt the natural acquisition of immunity [27]. Thus, in addition to seasonal reductions in tick abundance during dry periods [16,23], intensive tick-control practices may create temporary gaps in pathogen exposure, potentially delaying infection among susceptible cattle and contributing to heterogeneous serological profiles within herds [13]. However, this hypothesis could not be directly evaluated in the present study because the intensity and timing of acaricide use were not assessed longitudinally.

### 4.2. Enzootic Status of B. bigemina

Although *B. bigemina* presented the highest number of enzootically Stable herds among the evaluated pathogens, only six of the 32 farms (18.8%) exceeded the seroprevalence threshold required for enzootic stability. Therefore, enzootically Stable herds represented a minority of the evaluated population, indicating that even the most favorable epidemiological scenario observed in the present study was still insufficient to characterize widespread enzootic stability.

Nevertheless, the greater frequency of enzootically Stable herds observed for *B. bigemina* suggests that this parasite is maintained more efficiently under field conditions than *B. bovis* [25,28]. Its lower pathogenicity probably allows infected animals to survive and remain within the herd, increasing opportunities for continued transmission by *R. microplus* [24,28]. Consequently, farms experiencing sustained tick challenges are more likely to maintain antibody levels above the threshold required for enzootic stability [19]. This biological advantage probably explains why *B. bigemina* achieved the highest proportion of enzootically stable herds despite sharing the same biological vector as *B. bovis*.

The multivariable analysis corroborated these epidemiological findings by identifying breed, production type, and animal purchase as independent factors associated with the enzootic status of *B. bigemina*. Among these variables, dairy production exhibited the strongest association, increasing the odds of enzootically stable herd profiles by approximately 74-fold, whereas Zebu cattle and other breeds showed markedly lower odds than Holstein cattle. However, the wide confidence interval around the estimate for production type (OR = 74.51; 95% CI: 3.80–6760) indicates substantial uncertainty in the magnitude of this association and warrants cautious interpretation.

These findings are biologically coherent because specialized dairy systems commonly rely on Holstein genetics, a breed consistently recognized as more susceptible to *R. microplus* infestation than Bos indicus cattle [29]. Increased tick infestation results in more frequent contact with infected vectors, promoting repeated exposure to *Babesia* spp. and increasing the likelihood of maintaining antibody levels compatible with enzootic stability [22].

Animal purchase was associated with an approximately tenfold increase in the odds of more favorable enzootic profiles for *B. bigemina*. This association probably reflects the dynamic nature of herd immunity, since the movement of cattle between farms may introduce both previously exposed animals and infected ticks into recipient herds. Consequently, animal movement can modify parasite circulation and herd-level serological profiles, increasing the probability of reaching the seroprevalence threshold required for enzootic stability.

### 4.3. Enzootic Status of A. marginale

A contrasting epidemiological pattern was observed for *A. marginale*. Although molecular analyses previously demonstrated that this was the most frequently detected CTF pathogen in the studied cattle population [7], its serological profile did not reflect a comparable level of herd immunity. Despite presenting a seroprevalence value similar to that observed for *B. bigemina*, only three herds achieved enzootic stability (Figure 1), indicating that widespread circulation of the pathogen did not translate into homogeneous population immunity.

This apparent discrepancy probably reflects important differences in the transmission dynamics of bovine anaplasmosis. Unlike *Babesia* spp., which depend almost exclusively on repeated biological transmission by infected ticks, *A. marginale* can also be disseminated through intermittent mechanical routes, including contaminated instruments and hematophagous insects [30,31]. Although these transmission pathways favor pathogen circulation, they do not necessarily ensure the continuous and homogeneous exposure of young animals required for the establishment of enzootic stability at the herd level [19].

The multivariable model identified reuse of needles as the only independent factor associated with the enzootic status of *A. marginale*. Farms reporting needle reuse exhibited 97% lower odds of achieving enzootic stability (OR = 0.03), even after adjustment for potential confounding variables. This finding is biologically plausible because iatrogenic transmission occurs through sporadic management procedures rather than continuous exposure to infected vectors [32,33]. Consequently, although needle reuse may facilitate the dissemination of *A. marginale* among individual animals, it is unlikely to provide the repeated and homogeneous exposure necessary for the development of herd immunity and enzootic stability [34].

The biology of A. marginale may also contribute to this pattern. Following acute infection, cattle can remain persistently infected and serve as lifelong reservoirs of the pathogen, with recurrent but relatively low-level rickettsemia associated with antigenic variation [35,36]. Therefore, the presence of infected cattle within a herd does not necessarily imply frequent or homogeneous exposure of susceptible animals. This may be particularly relevant for mechanical transmission, whose efficiency depends strongly on the rickettsemia of the source animal [37]. Consequently, pathogen circulation may persist without providing the early and sufficiently uniform exposure required for enzootic stability.

### 4.4. Study Limitations

This study has some limitations that should be considered when interpreting the results. Although the sampling strategy was designed to reflect the predominantly small-scale herd structure of the study regions, only five or six animals were sampled per farm. Consequently, the observed within-herd seroprevalence has limited numerical resolution, particularly in smaller herds. This is especially relevant for the 12.5% threshold used in the enzootic classification, as a single seropositive animal can substantially change the observed proportion of seropositive animals in a small sample. Nevertheless, detection of even one seropositive animal provides evidence of previous exposure within the sampled herd and therefore does not support classification as non-immune.

The risk-factor analyses were based on 32 farms, and some explanatory-variable categories contained relatively few observations. This limited the number of farms available for estimating some associations and resulted in particularly wide confidence intervals for several risk estimates. For example, the association between dairy production and *B. bigemina* enzootic status showed an OR of 74.51 (95% CI: 3.80–6760), indicating substantial uncertainty regarding the magnitude of the estimated effect. Similar imprecision was observed for Animal Purchase with an OR of 10.43 (95% CI: 0.77–438.06), whereas the estimates for the protective associations were generally associated with narrower confidence intervals. Therefore, risk estimates with wide confidence intervals should be interpreted cautiously and considered exploratory.

An additional limitation is that the diagnostic performance of the commercial ELISA kits was not independently evaluated in the present study. Although the manufacturer reports sensitivity >83% and specificity >81% for the *A. marginale* assay, corresponding performance estimates were not provided in the available documentation for the *B. bovis* and *B. bigemina* assays. In addition, quantitative information regarding potential cross-reactivity between the assays was not available from the manufacturers. Therefore, species-specific serological results should be interpreted within the performance characteristics reported for the respective assays.

## 5. Conclusions

The present study demonstrated that the enzootic status of cattle tick fever pathogens differs substantially among pathogens, despite their circulation within the same cattle population. While *B. bigemina* presented the highest frequency of enzootically stable herds, stability remained uncommon, whereas no herd achieved enzootic stability for *B. bovis*. *Anaplasma marginale*, although previously identified as the most prevalent pathogen by molecular detection, also failed to establish homogeneous herd immunity, reinforcing that pathogen circulation alone does not necessarily result in enzootic stability.

Overall, the predominance of enzootically unstable and non-immune herds suggests that pathogen circulation alone may not be sufficient to establish a favorable enzootic profile under the epidemiological conditions of Paraíba State. Consequently, continuous epidemiological surveillance, appropriate herd management, and integrated vector control remain essential to reduce disease risk and support sustainable cattle production in the region.

## Figures and Tables

**Table 1:** Seroprevalence of antibodies against CTF pathogens according to climatic region.

Pathogen	Semi-Arid (%)	Tropical Humid (%)	Total (%)
*B. bovis*	12/90 (13.3)	12/85 (14.1)	24/175 (13.7)
*B. bigemina*	20/90 (22.2)	38/85 (44.7)	58/175 (33.1)
*A. marginale*	31/80 (38.7)	29/75 (38.6)	60/155 (38.7)
At least one pathogen	41/90 (45.6)	56/85 (65.9)	97/175 (55.4)

**Table 2:** Univariate and multivariate analysis of factors associated with the enzootic status of *Babesia bovis* in cattle farms from Paraíba State, Northeast Brazil.

**Univariate Analysis**			
**Variable**	**Category**	**OR (95% CI)**	***p*** **-Value**
Region	Tropical humid/Semi-arid	1.00 (0.24–4.10)	1.000
Age	2–5 years/0–2 years	0.18 (0.01–1.31)	0.241
	>5 years/0–2 years	0.03 (0.00–0.39)	0.021 *
Sex	Male/Female	NE^θ^	0.993
Breed	Holstein crossbred	1.27 (0.05–34.69)	0.239
	Zebu	1.00 (0.01–69.47)	1.000
	Other breeds	2.00 (0.04–120.28)	0.711
Production type	Beef/Dairy	1.50 (0.33–7.47)	0.606
Production system	Semi-intensive/Extensive	1.18 (0.12–11.23)	0.877
Shared pasture	Yes/No	3.82 (0.73–29.51)	0.138
Animal purchase	Yes/No	1.33 (0.33–5.61)	0.688
Bull rotation between farms	Yes/No	1.59 (0.36–7.66)	0.543
**Multivariate Analysis**			
**Variable**		**OR (95% CI)**	***p*** **-Value**
Age (2–5 years)		0.18 (0.01–1.42)	0.154
Age (>5 years)		0.03 (0.00–0.44)	0.025 ^●^

Abbreviations: OR = odds ratio; CI = confidence interval. NE^θ^: Not estimable due to complete separation. *: Statistically significant in the univariate analysis (*p* ≤ 0.2). ^●^: Statistically significant in the multivariable analysis (*p* ≤ 0.05).

**Table 3:** Univariate and multivariate analysis of factors associated with the enzootic status of *B. bigemina* in cattle farms from Paraíba, Brazil.

**Univariate Analysis**			
**Variable**	**Category**	**OR (95% CI)**	***p*** **-Value**
Region	Tropical humid/Semi-arid	2.77 (0.72–11.68)	0.146
Age	2–5 years/0–2 years	0.32 (0.06–1.53)	0.168 *
	>5 years/0–2 years	0.09 (0.01–0.80)	0.110 *
Sex	Male/Female	3.00 (0.45–22.04)	0.258
Breed	Holstein crossbred	0.19 (0.01–2.89)	0.232
	Zebu	0.07 (0.00–2.76)	0.172 *
	Other breeds	0.11 (0.00–3.17)	0.203
Production type	Beef/Dairy	7.57 (1.54–47.98)	0.019 *
Production system	Semi-intensive/Extensive	0.70 (0.09–5.47)	0.734
Shared pasture	Yes/No	1.62 (0.38–7.22)	0.519
Animal purchase	Yes/No	6.51 (1.53–35.52)	0.017 *
Bull rotation between farms	Yes/No	2.51 (0.62–11.07)	0.206
**Multivariate Analysis**			
**Variable**		**OR (95% CI)**	***p*** **-Value**
Region		2.48 (0.39–18.73)	0.344
Age (2–5 years)		0.04 (0.00–0.79)	0.068
Age (>5 years)		0.08 (0.00–2.84)	0.205
Breed (Holstein crossbred/Holstein)		0.04 (0.00–3.71)	0.174
Breed (Zebu/Holstein)		<0.001 (0.00–0.04)	0.020 ^●^
Breed (Other breeds/Holstein)		<0.001 (0.00–0.03)	0.006 ^●^
Production type		74.51 (3.80–6760)	0.017 ^●^
Animal purchase (Yes/No)		10.34 (1.36–143.33)	0.040 ^●^

Abbreviations: OR = odds ratio; CI = confidence interval. *: Statistically significant in the univariate analysis (*p* ≤ 0.2). ^●^: Statistically significant in the multivariable analysis (*p* ≤ 0.05).

**Table 4:** Univariate and multivariate analysis of factors associated with the enzootic status of *A. marginale* in cattle farms from Paraíba, Brazil.

**Univariate Analysis**			
**Variable**	**Category**	**OR (95% CI)**	***p*** **-Value**
Region	Tropical humid/Semi-arid	0.96 (0.20–4.47)	0.956
Age	2–5 years/0–2 years	1.00 (0.12–8.69)	1.000
	>5 years/0–2 years	0.08 (0.00–0.88)	0.051 *
Sex	Male/Female	1.75 (0.11–29.68)	0.691
Breed	Holstein crossbred	0.61 (0.01–30.13)	0.812
	Zebu	1.00 (0.01–102.71)	1.000
	Other breeds	0.18 (0.00–18.39)	0.471
Production type	Beef/Dairy	2.20 (0.40–14.31)	0.375
Production system	Semi-intensive/Extensive	0.70 (0.09–5.90)	0.732
Shared pasture	Yes/No	1.19 (0.23–6.39)	0.837
Animal purchase	Yes/No	4.86 (0.93–37.94)	0.082 *
Bull rotation between farms	Yes/No	11.19 (1.55–233.21)	0.039 *
Same needle use	Yes/No	0.09 (0.00–0.66)	0.040 *
Horn Flies	Yes/No	1.29 (0.27-6.67)	0.753
**Multivariate Analysis**			
**Variable**		**OR (95% CI)**	***p*** **-Value**
Age (2–5 years)		5.74 (0.44–154.90)	0.208
Age (>5 years)		0.59 (0.02–23.04)	0.753
Animal purchase (Yes/No)		1.65 (0.17–18.56)	0.665
Bull rotation between farms (Yes/No)		10.43 (0.77–438.06)	0.125
Same needle use (Yes/No)		0.03 (0.00–0.38)	0.019 ^●^

Abbreviations: OR = odds ratio; CI = confidence interval. *: Statistically significant in the univariate analysis (*p* ≤ 0.2). ^●^: Statistically significant in the multivariable analysis (*p* ≤ 0.05).

## Data Availability

The original contributions presented in this study are included in the article. Further inquiries can be directed to the corresponding author.
